# Features of diaphragmatic myositis in a case of sudden infant death

**DOI:** 10.3109/03009734.2011.588347

**Published:** 2011-06-29

**Authors:** Michael Eisenhut

**Affiliations:** Luton & Dunstable Hospital NHS Foundation Trust, Luton, United Kingdom

Dear Sir,

A recent review article (1) was very helpful in understanding the cause of death of a patient recently witnessed. This patient however also showed differences to what was reported in this review. The 5-month old infant was admitted with features of a viral respiratory tract infection and poor feeding, and an unexpected respiratory arrest 2 days after admission led to a sudden death in hospital. A post-mortem and microbiological work-up revealed the presence of rhinovirus in respiratory secretions and adenovirus in the gut. The diaphragm was macroscopically normal but on histology showed focal infiltrate of macrophages and small lymphocytes forming linear aggregates between myofibre bundles, with evidence of myofibre destruction, focal segmental myocyte necrosis, and myocyte regeneration. Myocardium, brain, and other skeletal muscle tissue showed no cellular infiltration. The inflammation observed may have weakened the diaphragm in our case, leading to respiratory arrest. The cause of the diaphragmatic myositis in our case may have been adenovirus which has previously been associated with myositis in children (2).

The authors of the review (1) emphasized that temporary diaphragmatic weakness in an infection-induced inflammatory response has not been associated with histologic abnormalities previously. A mechanism may be weakening of electromechanical coupling by inflammatory mediators. The skeletal muscle ryanodine receptor Ca^2+^ release channel is a key component of the excitation–contraction coupling machinery in striated muscle. Nitric oxide produced by the cytokine- and endotoxin-induced inducible nitric oxide synthase during inflammatory processes influences the contractility of skeletal muscles (3). *In vivo*, the initial release of NO activates ryanodine receptors, but a higher concentration of NO inhibits ryanodine receptors. NO alters ryanodine receptor binding activity by S-nitrosylation or oxidation of several classes of cysteine residues associated with ryanodine receptor protein (3). Influence of inflammatory mediators on ion channel activity may also explain the potentially clinically important phenomenon of diaphragmatic flutter which has been associated with respiratory syncytial virus (RSV) infection and difficulties with weaning patients from the ventilator (4).

Future research needs to concentrate on case control studies, comparing the histology of diaphragms in infants dying from sudden infant death and death from other causes during the same season of the year to allow for the influence of seasonal viral infections. The degree of nitrosylation of cellular proteins should be compared between groups. Specific electrophysiological studies should be employed to assess the contribution of diaphragmatic flutter to apneas associated with viral respiratory tract infections in young infants.
